# Jianpi Zishen Decoction ameliorates renal damage induced by systemic lupus erythematosus through inhibition of the TLR4/MAPK pathway

**DOI:** 10.1080/13880209.2025.2606959

**Published:** 2025-12-29

**Authors:** Zhongfu Tang, Lili Cheng, Ming Li, Junjie Chen, Chuanbing Huang

**Affiliations:** Department of Rheumatology, The First Affiliated Hosppital of Anhui University of Traditional Chinese Medicine, Hefei, China

**Keywords:** Systemic lupus erythematosus, kidney damage, Jianpi Zishen Decoction, immunoinflammatory disorders

## Abstract

**Context:**

Systemic lupus erythematosus (SLE) is an autoimmune disease involving multiple systems. Jianpi Zishen Decoction (JZD) is a TCM formula used to improve proteinuria in SLE, which has been widely used in the treatment of renal damage in SLE.

**Objective:**

To investigate the potential targets and action mechanisms of JZD in ameliorating renal damage in SLE through network pharmacology and *in-vivo* and *in-vitro* experiments.

**Methods:**

The main components of JZD were determined using UPLC-Q-TOF-MS/MS. The potential targets and action mechanisms of JZD were explored using network pharmacology and molecular docking techniques. MRL/lpr mice were used as animal models, and their renal pathological changes were observed by HE staining, periodic acid-Schiff staining, and Masson staining. Additionally, mouse glomerular ultrastructure was observed using TEM. The level of urinary protein, immunoinflammatory indicators, and TLR4/MAPK pathway-related molecules were detected through a variety of experimental methods. Furthermore, the effects of JZD on LPS-stimulated HMCs were evaluated.

**Results:**

A total of 27 prototype components were identified in the blood-entered component of JZD. Animal experiments showed that JZD effectively ameliorated renal damage and immune disorders, reduced glomerular scores and vascular wall scores, as well as attenuated IgG and C3 deposition in the kidneys of MRL/lpr mice. Network pharmacological analysis and molecular docking identified TLR4 as a core potential target of JZD’s efficacy. JZD inhibited the expression of TLR4, p38, JNK, AP-1, and pro-inflammatory factors in the kidneys of MRL/lpr mice. *In-vitro* experiments further showed that JZD inhibited LPS-induced HMC proliferation.

**Conclusion:**

JZD could ameliorate SLE renal damage and its active ingredients exerted therapeutic effects by inhibiting the TLR4/MAPK pathway, highlighting the therapeutic potential of JZD in modulating immunoinflammation.

## Introduction

Systemic lupus erythematosus (SLE) is a chronic autoimmune disease where renal involvement represents the primary cause of poor prognosis. Global epidemiological data show an SLE prevalence of 15.87–108.92 per 100,000 population (Tian et al. 2023), with urban China reporting rates of 41.77–53.83 per 100,000. Notably, women exhibit a 5.27-fold higher incidence than men, particularly during childbearing years (M. Li et al. 2024). Renal involvement occurs in about 50% of SLE patients, which may progress into lupus nephritis (LN), and even lead to end-stage renal disease (Perge et al. 2025). It is also a major contributor to disability and death in SLE (Kumar et al. 2025). Currently, glucocorticoids and immunosuppressive agents are mainly used methods in the clinic to control disease progression; however, long-term drug use is generally accompanied by risk of infections and organ toxicity and it is difficult to avoid (Belmont 2025). Therefore, investigating effective intervention strategies and mitigating the toxic side effects of drugs have been the focus of research in the field of SLE.

The pathogenesis of SLE is complex and involves interactions among genetic susceptibility, environment, immune system dysregulation, and epigenetic modifications (Accapezzato et al. 2023). In SLE, glomerular immune complex deposition triggers complement-mediated inflammation, driving lupus nephritis and renal damage (Roveta et al. 2024). The clinical priority for treating SLE with renal damage is to reduce renal impairment. A randomized clinical trial indicated that Mizoribine can serve as induction therapy for LN. Compared to intravenous cyclophosphamide, patients better tolerate oral Mizoribine (Dong et al. 2025). In a clinical study of 8 refractory SLE patients, belimumab-rituximab combination therapy induced long-term remission in 6 cases (75%) (van Schaik et al. 2025). Although biologics have stronger targeting ability, their high cost and individual differences in efficacy limit their clinical application.

Current clinical evidence indicates that TCM has the unique advantages of ‘multi-component and multi-target’ synergy in regulating SLE immune homeostasis (Dou et al. 2024; Liu et al. 2022). A retrospective study of 10,462 SLE patients demonstrated that combining TCM with conventional treatment markedly reduced proteinuria and mortality risk (Wei et al. 2025). Additionally, a meta-analysis of 14 studies concluded that TCM effectively reduced disease activity, improved patient prognosis, and decreased the incidence of adverse events from Western medicine (J. Li et al. 2024). Jianpi Zishen decoction (JZD) is a classic TCM prescription formulated by our team based on TCM theory to intervene in SLE with renal damage. Currently, JZD has been widely used in clinical practice and shows renal protective effects (Shang et al. 2022). However, its specific mechanism remains unclear.

MRL/lpr mice are a classic spontaneous animal model for SLE research, which can highly mimic human SLE multiorgan damage and have been commonly used in experimental animal studies of SLE (Reynolds et al. 2024). Lipopolysaccharide (LPS), a major component of the outer membrane of Gram-negative bacteria, is a natural activator of Toll-like receptor 4 (TLR4) and is used to stimulate human glomerular mesangial cells (HMCs) to establish *in vitro* models (L. Chen et al., 2024). The present study investigates the possible mechanism of JZD in ameliorating renal damage in SLE through network pharmacology, molecular docking, and *in-vivo* (using MRL/lpr mice as an animal model) and *in-vitro* [LPS-induced simulated renal damage in HMCs] experiments.

## Materials and methods

### Experimental animals

MRL/lpr mice represent a classic spontaneous model for SLE research. 40 MRL/lpr female mice and 8 C57BL/6 female mice (7 weeks, 18-22 g) were purchased from Shanghai SLAC Laboratory Animal Co., Ltd. [Shanghai, China, license number SCXK (Shanghai) 2022-0004]. All animals were housed (25 ± 2 °C, 50 ± 10% humidity) in the Animal Experiment Platform of the Institute of Artificial Intelligence, Hefei Comprehensive National Science Center under specific pathogen-free (SPF) conditions at a 12 h light/dark cycle, with free drinking water and food provided. All mice were adaptively fed for 1 week before the experiment. All animal experiments were approved by the Animal Ethics Committee of Anhui University of Traditional Chinese Medicine (Approval No.: AHUCM-mouse-2022130) and conducted in accordance with the Guide for the Care and Use of Laboratory Animals and the ARRIVE guidelines (https://arriveguidelines.org/).

### Preparation of JZD and analysis of blood-entered components

JZD comprises 8 herbs, as indicated in Table 1. The batch numbers of the 8 herbs are as follows: Astragali Radix: 240518; Rehmanniae Radix Preparata: 240806; Poria: 2309112; Atractylodis Macrocephalae Rhizoma: 240525; Rhizoma Dioscoreae: 2403019; Cuscutae Semen: 240810; Rubi Fructus: 2401150; Rosae Laevigatae Fructus: 240716. These herbs were purchased from Anhui Purun Traditional Chinese Medicine Extracts Co., Ltd. (Bozhou, China). In short, the herbs were added with distilled water at 8 times the volume of the herb, soaked, and decocted for 30 min. After that, the liquid was poured out, and distilled water at 5 times the volume of the herb was added, followed by decoction for 30 min. Next, two decoctions were combined and the residue was filtered off. Finally, the liquid was concentrated into a liquid containing 1.8 g/mL raw drug and then stored at 4 °C for spare use. The recommended clinical dose is 100 g/d for a 70 kg adult, and the equivalent dose for mice is calculated to be 13 g/kg/d and for rats is 9 g/kg/d based on the surface area of the animal and the human body.

**Table 1.** Composition of JZD.

Botanical name	Chinese name	Plant part used	Dosage used
Astragali Radix	Huangqi	Root of *Astragalus membranaceus var. mongholicus*, Fabaceae	15 g
Rehmanniae Radix Preparata	Shudihuang	Processed root of *Rehmannia glutinosa*, Orobanchaceae	10 g
Poria	Fuling	Sclerotium of Poria cocos, Polyporaceae	12 g
Atractylodis Macrocephalae Rhizoma	Baizhu	Rhizome of *Atractylodes macrocephala*, Asteraceae	10 g
Rhizoma Dioscoreae	Shanyao	Rhizome of *Dioscorea opposita Thunb*., Dioscoreaceae	15 g
Cuscutae Semen	Tusizi	Seed of *Cuscuta chinensis*, Convolvulaceae	15 g
Rubi Fructus	Fupenzi	Immature fruit of *Rubus chingii*, Rosaceae	15 g
Rosae Laevigatae Fructus	Jinyingzi	Fruit of *Rosa laevigata*, Rosaceae	8g

Ultra-performance liquid chromatography quadrupole time-of-flight tandem mass spectrometry (UPLC-Q-TOF-MS/MS) coupled with the UNIFI database was used to analyze JZD’s blood-entered components in rat serum samples after gavage. Briefly, the whole blood of rats after JZD gavage was taken, and centrifuged (3000 r/min, 10 min) after standing at room temperature for 1 h, with the supernatant taken. The separation was performed on a Waters ACQUITY UPLC BEH C18 column (2.1 mm × 100 mm, 1.7 μm), with the mobile phase of 0.1% formic acid in water (A)-acetonitrile (B). The elution gradient conditions were 0-3 min, 10%-16% B; 3-6 min, 16%-20% B; 6-12 min, 20%-25% B; 12-17 min, 25%-35% B; 17-21 min, 35%-45% B; 21-24 min, 45%-60% B; 24-28 min, 60%-80%B; 28-30 min, 80%-90%B; 30-32 min, 90%-10%B, and 32-34min, 10%B. The flow rate was 0.2 mL/min, the column temperature was 35 °C, and the injection volume was 2 μL. The mass spectrometry scanning range was set from 50 to 1200 m/z, and the detection was carried out using electrospray ionization (ESI) in positive and negative ion modes, respectively, with leucine enkephalin as the accurate mass number calibration solution. The volume flow rate of nebulizing gas (nitrogen) was 600 L/h, the desolvation temperature was 350 °C, the cone-aperture gas flow rate was 50 L/h, the ion source temperature was 110 °C (ESI-) and 120 °C (ESI+), the cone-aperture voltage was 40 V, and the ion-spray voltages were 2.5 KV (ESI-) and 3.0 KV (ESI+). For MSE scanning mode detection, the collision voltage for the low-energy channel was 6 V and for the high-energy channel was 20-80 V. Masslyxn 4.1 software was used for the identification of blood-entered components in combination with the retention time of the control product and literature reports.

### Experimental groups and interventions

A total of 40 MRL/lpr mice were randomly allocated into the MRL/lpr group (treated with the same volume of normal saline by gavage), prednisone group [pred; treated with prednisone suspension (5 mg/kg/d) by gavage], JZD low-dose group (JZD-L), JZD medium-dose group (JZD-M), and JZD high-dose group (JZD-H) [treated with JZD solution (13 g/kg/d, 26 g/kg/d, and 52 g/kg/d, respectively) by gavage], with 8 mice in each group, with 8 C57BL/6 mice used as the control group (treated with the same volume of normal saline by gavage). The mice in each group received gavage once a day for 8 weeks. During the intervention period, mouse urine was collected and mice were weighted. After 8 weeks of administration, mice were anesthetized by intraperitoneal injection of 3% pentobarbital sodium (Sigma-Aldrich, P3761). Blood samples were collected from the retro-orbital plexus. About 1 ml of blood was collected from each mouse. Subsequently, euthanasia was performed *via* carbon dioxide (CO_2_) inhalation. The initial gas flow rate was maintained at 30-70% chamber volume displacement per minute (typically 1-2 L/min for standard cages). Upon loss of consciousness, the flow rate was reduced to 10-30% displacement (0.5-1 L/min) to minimize distress while ensuring vital function cessation. Death was confirmed by absence of respiration and corneal reflex. Renal tissues were immediately harvested for subsequent analysis.

### Preparation of JZD-contained serums (JZD-S)

A total of 20 male SD rats (3 weeks) were randomly allocated into the JZD group (*n* = 10, administered by gavage at five times the equivalent dose for adults in the clinic, i.e., 45 g/kg/d) and blank group (*n* = 10, administered by gavage the equivalent amount of saline). The two groups of rats were gavaged twice a day at intervals of 12 h for 7 consecutive days. After the last gavage for 1 h, blood was collected from the abdominal aorta under anesthesia with 1% sodium pentobarbital and centrifuged (3000 r/min, 15 min). The supernatant was collected and combined with the serum of the same group, which was placed in a water bath (56 °C) for inactivation for 30 min, and then filtered (0.22 μm filter), sterilized, and stored at −80 °C. Before use, the RPMI-1640 culture medium (Wuhan Pricella, PD003) was added, and the medium with the corresponding concentration of JZD-contained serum was prepared according to the requirements for subsequent cell experiments.

### Cell culture

HMCs (Lonza, CC-2547, CVCL_2685) were grown in DMEM/F12 medium containing 10% fetal bovine serum (FBS) (New Zealand, Cytiva, SH30406.05) and 1% penicillin-streptomycin (NCM Biotech, C100C5) in an incubator (37 °C, 5% CO_2_) for continuous cultivation. When the cells grew to 80%-90% confluence, they were passaged and frozen, and the 3^rd^-10^th^ generations of HMCs were selected for the experiments. The optimal induction concentration and action time of LPS (Xinbosheng Biotechnology, GC2059) were screened. Briefly, HMCs were induced with 5 μM, 10 μM, 15 μM, 20 μM, and 50 μM of LPS for 0 h, 12 h, 24 h, 48 h, and 72 h, respectively, and the cell viability of each group was detected using cell counting kit-8 (CCK-8) assay. Meanwhile, the optimal serum concentration and action time of JZD were screened. Specifically, 5%, 10%, and 20% of JZD serum were used to act on HMCs for 0 h, 12 h, 24 h, 48 h, and 72 h, respectively, and the cell viability of each group was detected using CCK-8 assay. The cells were grouped into the blank serum group (control), LPS induction group (LPS), and LPS + JZD optimal serum (LPS + JZD-S) group.

### CCK-8 assay

Cell viability was detected using the CCK-8 kit (Biosharp, BS350B). The cells were collected and made into cell suspension. Cell concentration was adjusted to 5-10 × 10^4^/mL by cell counting. After that, the cell suspension was gently mixed, and then added (100 μL) to each well, with the edge wells filled with sterile phosphate-buffered saline (PBS) (Cytiva, SH30256.01). The seeded cell culture plates were placed in an incubator and cultured until the cell monolayer was spread to the bottom of the wells (96-well plates). After overnight incubation in an incubator (37 °C, 4.5% CO_2_), cells were observed under an inverted microscope (Olympus, CKX31). After incubation for different times, 10 μL CCK-8 solution was added to each well, followed by further culture for 1-4 h. The optical density (OD) value of each well was measured at a wavelength of 450 nm utilizing enzyme-linked immunosorbent assay (ELISA). Blank wells (medium and CCK-8 solution) were set at the same time. Cell viability = [(experimental wells − blank wells)/(control wells -blank wells)].

#### 5-ethynyl-2’-deoxyuridine (EdU) staining

Cell proliferation was detected utilizing EdU kits (Shanghai Yaze Biotechnology, CX002). After the cells were well grown on the culture plate, the medium was aspirated and slowly washed 3 times with PBS-T, 3 min each time. The plates were fixed with 2.4% paraformaldehyde (Beijing Solab, P1110) for 20 min and slowly washed 3 times with PBS-T, 3 min each time. After that, 0.5% TritonX-100 was added dropfold for incubation (37 °C, 30 min) in an incubator. The slides were then removed and sealed with an anti-fluorescence quench sealing agent. A fluorescence microscope was employed for observation and photographing, with green fluorescence for the EdU signal and blue fluorescence for nuclear staining.

### Enrichment analysis

The GSE154851 dataset was downloaded from the GEO database (https://www.ncbi.nlm.nih.gov/geo/). Differential gene expression analysis was performed by comparing samples from 38 SLE patients (GSM4681537-GSM4681574) and 32 healthy individuals (GSM4681575-GSM4681606). Differentially expressed genes (DEGs) were screened with|*log*2FC|> 2 and *p*-value < 0.05; 719 DEGs were obtained and volcano maps and heat maps were generated. Next, the screening criteria were set as |*log*2FC| > 4 and *p*-value < 0.01 to further narrow the scope of the DEGs, yielding 112 DEGs. These DEGs were subjected to Gene Ontology (GO) enrichment analysis using the Metascape platform (https://metascape.org/gp/index.html). Gene set enrichment analysis (GSEA) was performed on all genes in the GSE154851 dataset, with the significance criteria set as normalized enrichment score (NES) > 2 and *p*-value < 0.05, and pathways unrelated to this study excluded.

### Network pharmacology and molecular docking

The blood-entered components of JZD were analyzed by UPLC-MS/MS, and the prototype compounds of JZD were determined through databases and relevant literature. Moreover, compound targets were predicted using the Swiss Target Prediction database (http://www.swisstargetprediction.ch/) and the HERB database (http://herb.ac.cn/). SLE-related DEGs were screened based on the GEO database. The intersection between JZD potential targets and SLE-related genes was visualized using the Venn diagram, and a network diagram was constructed using Cytoscape software. After that, the intersection targets were imported into the STRING database to construct a protein-protein interaction (PPI) network and screen the core targets. Meanwhile, the intersection targets were subjected to GO and Kyoto Encyclopedia of Genes and Genomes (KEGG) enrichment analyses.

Network pharmacology results revealed that TLR4 was a potential target of JZD. Hence, the molecular structure of TLR4 was downloaded from the PDB database (https://www.rcsb.org/). Additionally, the 3D structure of formononetin, isoacteoside, diosmetin, isorhamnetin, and atractylenolide III was downloaded from the PubChem database (https://pubchem.ncbi.nlm.nih.gov/). Molecular docking was performed using AutoDock Vina software to explore six possible binding sites. PyMOL software was used to visualize the docking results and the binding energy was displayed using a heat map to evaluate the binding ability of ligands and receptors.

### Renal histopathology

Kidney tissues were fixed in 4% paraformaldehyde (Beijing Solab, P1110) for 48 h and then rinsed with running water. After dehydration using 75%, 85%, 90%, and 95% ethanol sequentially, tissues were placed in xylene for transparency, paraffin-embedded, sectioned, and then deparaffinized.

Hematoxylin-eosin (HE) staining was conducted. Specifically, kidney tissue sections were submerged in hematoxylin (Ebiogo, B006) for 5 min and washed with distilled water. The sections were treated with saturated lithium carbonate solution until sections turned blue and then washed again. After dehydration in 95% ethanol (Shanghai Guangnu Chemical, 20241109) for 2 min, the sections were submerged in eosin solution for 3-min staining and washed again. Afterward, the sections were placed in anhydrous ethanol for 5 min for dehydration, and this was repeated 3 times; the sections were then put into xylene for 5 min for transparency, and this was repeated 2 times, followed by sealing with neutral gum. Furthermore, periodic acid-Schiff staining (PAS) was also conducted. After that, the sections were immersed in Schiff’s reagent (Ebiogo, B017) for 10 min, washed with distilled water, dehydrated, cleared, and sealed with neutral gum. As for Masson staining, the sections were first stained with iron hematoxylin staining solution (Ebiogo, B022) for 8 min. Next, the sections were stained with aniline blue solution for 5 min, dehydrated, cleared, and sealed with neutral gum (Ebiogo, B018). A light microscope was employed for observations and recording.

### Observation of glomerular ultrastructure by a transmission electron microscope (TEM)

The kidney tissues were subjected to primary fixation, secondary fixation, gradient ethanol dehydration, resin infiltration, and embedding, and then ultrathin sections were prepared. The sections were stained with aqueous uranyl acetate (Servicebio, G106) for 30 min and lead citrate for 5 min and then rinsed thoroughly with distilled water to avoid crystallization of the stain. The glomerular structure was observed and recorded using TEM (Japan electronics, JEM1400).

### Biochemical kit testing

Mouse urine was collected, and 24-hour urine protein quantification (24hPRO), total protein, albumin, and creatinine were detected using a urine protein quantification kit, urine albumin kit, urine protein kit, and urine creatinine kit, respectively. The urine total protein-to-creatinine ratio (UTPCR) and urine albumin-to-creatinine ratio (UACR) were calculated. The biochemical kits were provided by Nanjing JianCheng BioEngineering Institute (Nanjing, China), and the specific operation process was referred to the kit’s protocol. The corresponding catalog numbers were: C035-2-1, A028-2-1, A045-4.

### ELISA

Mouse blood was collected and centrifuged to obtain the serum. ELISA kits (Wuhan JiYinmei Technology Co.) were used to detect the level of anti-double-stranded DNA antibody (anti-dsDNA), IgG, C3, and C4 in serum as per the kit’s protocol. The corresponding catalog numbers were: JYM1061Mo, JYM0031Mo, JYM0293Mo, JYM0268Mo.

### Real-time quantitative polymerase chain reaction (RT-qPCR)

The kidney tissue was ground into powder in liquid nitrogen, or cell precipitate was collected and lysed by adding 1 mL TRIzol (Life technogies, 15596018CN) for stratification and RNA precipitation to complete RNA extraction. RT reaction was performed with the following reaction procedure: 65 °C for 5 min (RNA denaturation), cooling for 2 min, 42 °C for 30 min (reverse transcription), and 70 °C for 5 min (inactivation of the enzyme) to obtain cDNA. The obtained cDNA was stored at −80 °C for subsequent experiments. The RT-qPCR reaction was conducted using a PCR instrument, and the relative expression of mRNA was calculated using the 2^-△△Ct^ method, with GAPDH as an internal reference. Primers were synthesized by Anhui ZhongKang Bio-technology Co., Ltd. (Anhui, China) (Table 2).

**Table 2.** Primers used for RT-qPCR.

Gene name	Species	Forward	Reverse
TLR4	Human	GCCACATGTCAGGCCTTATG	TTGGTTGAAATGCCCACCTG
	Mouse	GTTCTCTCATGGCCTCCACT	TTGGTTGAAATGCCCACCTG
p38	Human	CTCATTAACAGGATGCCAAGC	CTTGGGCCGCTGTAATTCTC
	Mouse	CCCCAGAGATCATGCTGAAT	CTTGGGCCGCTGTAATTCTC
JNK	Human	GGCAGCGTCTCTGTTACTCA	CAGCGAACGGCAACAGAAAA
	Mouse	GCATGGTGGTGGTTGTTTCT	TCTGTTGGACACCTGGAGAC
ERK1	Human	TCAACACCACCTGCGACCT	CGTAGCCACCTGCGACCT
	Mouse	TGGACATCACCAGCTTCTCC	AAGTTGCTGCTGGTGATGGT
AP-1	Human	GCTGAGCCTACAGATGAACT	GGCAGGATACCCAAACAAAC
	Mouse	TTGAGCTCAGGCTGGATAAGT	GGCAGGATACCCAAACAAAC
GAPDH	Human	TTCCACCCATGGCAAATTCC	ATCTCGCTCCTGGAAGATGG
	Mouse	GCAGTGGCAAAGTGGAGATTG	ATCTCGCTCCTGGAAGATGG

### Western blotting

Kidney tissue or cell samples were collected, lysed by adding 600 µL of radio immunoprecipitation assay (RIPA) (Beyotime, P0013B) cell lysate, and centrifuged (12000 r/min, 15 min), with the supernatant collected to obtain total tissue protein. After blocking for 2 h at room temperature on a shaker, the membranes were incubated (4 °C, overnight) with primary antibodies: TLR4 (Bioss, bs-20594R, RRID: AB_11162364, 1:1500), p38 (Bioss, bs-55529R, RRID: AB_11162538, 1:2000), p-p38 (Abcam, ab195049, RRID: AB_331641, 1:2000), JNK (Santa Cruz, sc-74532, RRID: AB_1121764, 1:2500), p-JNK (Abcam, ab156425, RRID: AB_331659, 1:2500), AP-1 (Bioss, bs-0670R, RRID: AB_10857132, 1:1500), ERK1 (Bioss, bs-1184R, RRID: AB_11109589, 1:1500) and GAPDH (Zsbio, TA-08, RRID: AB_2747419, 1:2000) with slow shaking. Following 3 washes with TBST, the membranes were added with HRP-labeled secondary antibody (Zsbio, ZB-2305, 1:20000) for incubation (room temperature, 1.2 h), and then rinsed 3 times with TBST. The membranes were developed with an enhanced chemiluminescence (ECL) kit (Biosharp, BL520A) and visualized using the chemiluminescence imaging system. Image J software was used for gray-level analysis.

### Immunofluorescence staining

Kidney tissue sections were deparaffinized in xylene (Tianjin KaiTong Chemical Company, T1256), followed by antigen retrieval. Next, the sections were added with 0.3% Triton X-100 (Ebiogo, B025) and permeabilized and blocked at room temperature. Afterward, the primary antibodies [TNF-α (Santa, sc-52746, RRID: AB_1123674, 1:200), IL-6 (Santa, sc-28343, RRID: AB_627494, 1:200), IL-12 (Bioss, bs-0767R, RRID: AB_11110819, 1:300), IgG (Affinity, DF13226, RRID: AB_2837415, 1:500), and C3 (Affinity, DF13224, RRID: AB_2837412, 1:200)] were added drop by drop for incubation (37 °C, 60 min). Next, a proper amount of HRP-labeled secondary antibody (RecordBio, RCB054) was added and incubated (room temperature, 30 min) in the dark. Afterward, tyramide signal amplification (TSA) fluorescent dye was added for incubation (room temperature, 10 min) in the dark, followed by 3 washes with PBS (Cytiva, SH30256.01). Sections were counterstained by dropping appropriate DAPI staining solution(Ebiogo, B028), incubated at room temperature for 5 min, and then washed with PBS for 5 min. The sections were sealed with an anti-fluorescence quenching agent, and a fluorescence microscope (Olympus, CX41) was employed for observation and image collection.

### Immunohistochemical staining

Kidney tissue sections were incubated (room temperature, 10 min) in 3% H_2_O_2_, and rinsed 3 times with PBS (Cytiva, SH30256.01). Next, 5% bovine serum albumin (BSA) (Bioss, bs-0370R) was added dropwise and the sections were sealed at room temperature for 30 min. After that, the primary antibodies [TLR4 (Bioworld, BS3489, RRID: AB_2687944, 1:200), p38(Bioss, bs-28027R, RRID: AB_11162543, 1:200), and JNK (Bioworld, BS1544, RRID: AB_2687932, 1:100)] were added dropwise for incubation (37 °C, 60 min). Next, HRP-labeled goat IgG (RecordBio, RCB056) was added for incubation (37 °C, 30 min). After rinsing with distilled water, the sections were re-stained with hematoxylin (Ebiogo, B006) for 2 min and differentiated with 1% hydrochloric acid alcohol for a few seconds, followed by bluing with lithium carbonate solution (Ebiogo, B011) for 30 s. Afterward, the sections were dehydrated, cleared with xylene, sealed with neutral gum, and observed under a microscope (Olympus, CX41).

### Statistical analysis

GraphPad Prism 9.0 software was applied for statistical analysis and data visualization. The previous sample size calculation by the research group showed that *n* ≥ 8, and the sample size for this study was determined to be 8. The measurement data were expressed as mean ± standard deviation, and one-way analysis of variance (ANOVA) was used for comparisons between groups that conformed to normal distribution, and non-parametric tests were used for those that did not conform to normal distribution. To account for multiple comparisons, we applied the Bonferroni correction for post-hoc tests following ANOVA. For non-parametric tests, the Dunn’s multiple comparison test with a Bonferroni adjustment was used. A *p*-value of less than 0.05 indicated a statistically significant difference.

## Results

### JZD ameliorated renal damage and immune disorders in MRL/lpr mice

**Figure 1.**
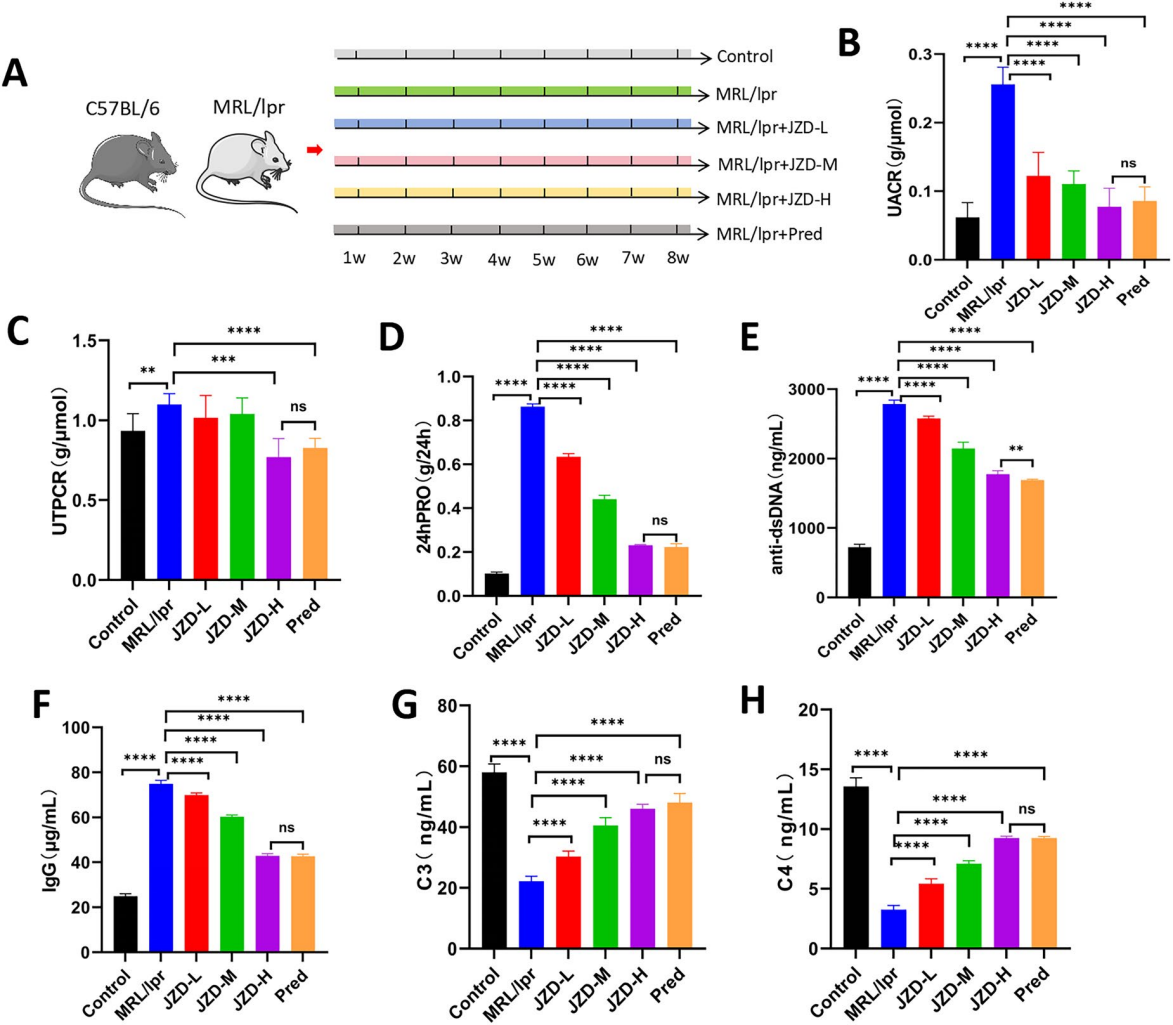
JZD ameliorates renal damage and immune deranges in MRL/lpr mice. (a) Overall schematic of the *in-vivo* experiment, with the light gray columns representing the blank group, green columns representing the MRL/lpr group, blue columns representing the JZD-L group, pink columns representing the JZD-M group, yellow columns representing the JZD-H group, and gray columns representing the prednisone group. (b–d) The 24hPRO, urinary total protein, urinary albumin, and urinary creatinine levels were detected by biochemical kits, and the UACR and UTPCR were calculated. (e–h) The serum levels of anti-dsDNA, IgG, C3, and C4 in each group were detected by ELISA. **p < 0.01, ***p < 0.001, ****p < 0.0001.

**Figure 2.**
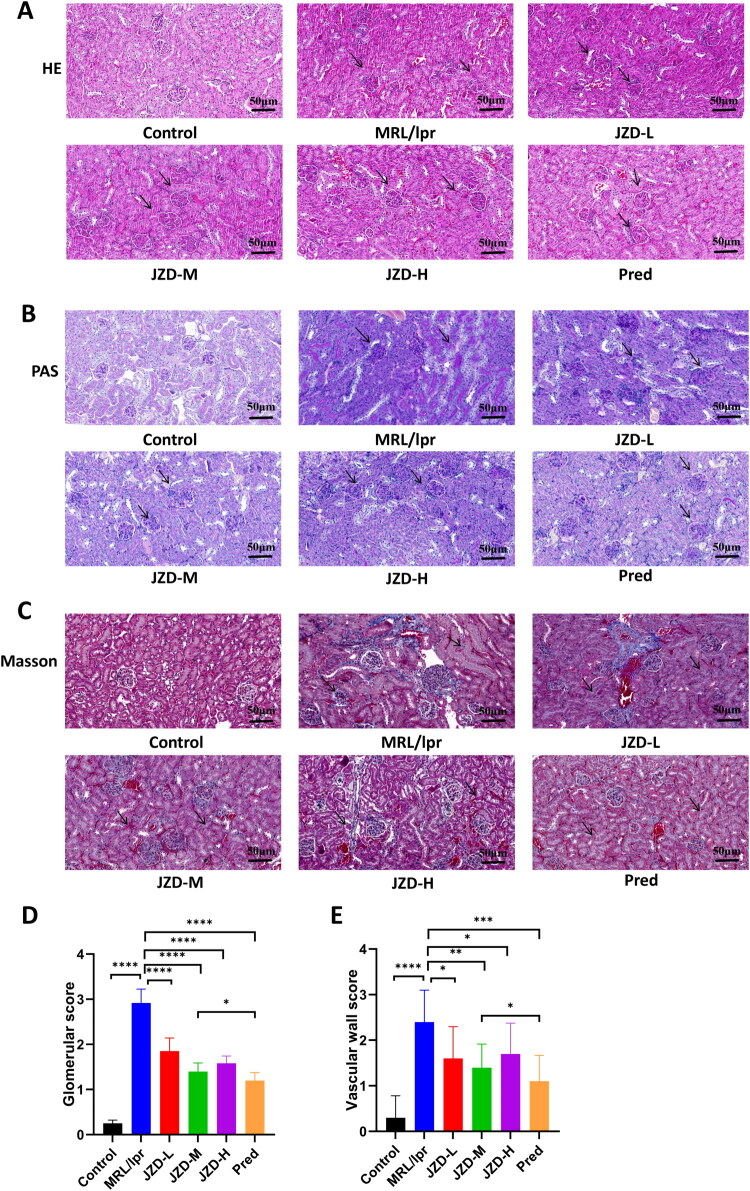
HE staining, PAS staining, and masson staining (20×, 50 μm) of kidney tissue sections were conducted. (a) HE staining of kidney tissue sections. (b) PAS staining of kidney tissue sections. (c) Masson staining of kidney tissue sections. (d) Glomerular scores of mice in each group. (e) Renal vessel wall scores of mice in each group. *p < 0.05, **p < 0.01, ***p < 0.001, ****p < 0.0001.

After 8 weeks of drug intervention in the six groups of mice (Figure 1(a)), the whole blood and urine of mice in each group were collected, and the protein levels in urine were detected using biochemical kits. The results showed that MRL/lpr mice had notably increased UACR, UTPCR, and 24hPRO compared with the control mice. After the intervention, the prednisone group, and the JZD-L, -M, and -H groups exhibited significantly reduced UACR, UTPCR, and 24hPRO, and there were no significant differences in any of these measures between the JZD-H group and the prednisone group (Figure 1(b–d), *p* < 0.05). Additionally, the levels of anti-dsDNA, IgG, C3, and C4 in mouse serum were detected by ELISA kits. As indicated by the results, MRL/lpr mice showed remarkably higher anti-dsDNA antibody and IgG levels and lower C3 and C4 levels than the control mice, while treatments in the JZD-L, -M, and -H groups, and the prednisone group caused opposite results, as manifested as significantly increased C3 and C4 levels and reduced anti-dsDNA antibody and IgG levels compared with the MRL/lpr group (Figure 1(e–h), *p* < 0.05). Moreover, HE staining (Figure 2(a)) revealed that the MRL/lpr group showed obvious capillary cell proliferation and mesangial proliferation, basement membrane thickening, accompanied by a large number of lymphocyte infiltration. Compared with the MRL/lpr group, the JZD-L, -M, and -H groups and the prednisone group showed alleviated cell proliferation and mesangial thickening, as well as reduced lymphocyte infiltration. As revealed by PAS staining (Figure 2(b)) results, there was obvious basement membrane thickening and more crescent formation in the MRL/lpr group, which could be effectively alleviated by intervention with the low-, medium-, and high-dose JZD and prednisone. Masson staining (Figure 2(c)) revealed that MRL/lpr mice showed obvious fibrosis in the glomerular capillary lumen, along with obvious thickening of the basement membrane and some global sclerosis. This phenomenon was markedly attenuated in the JZD-L, -M, and -H groups and the prednisone group, with the prednisone group showing the least severe fibrosis. Compared with the MRL/lpr group, the glomerular scores and vascular wall scores in the low-, medium-, and high-dose JZD groups were significantly decreased, and the degree of kidney injury was alleviated (Figure 2(d, e), *p* < 0.05). These results suggested that both JZD and prednisone could ameliorate renal damage and immune disorders in MRL/lpr mice.

### JZD attenuated IgG and C3 deposition in the kidneys of MRL/lpr mice

**Figure 3.**
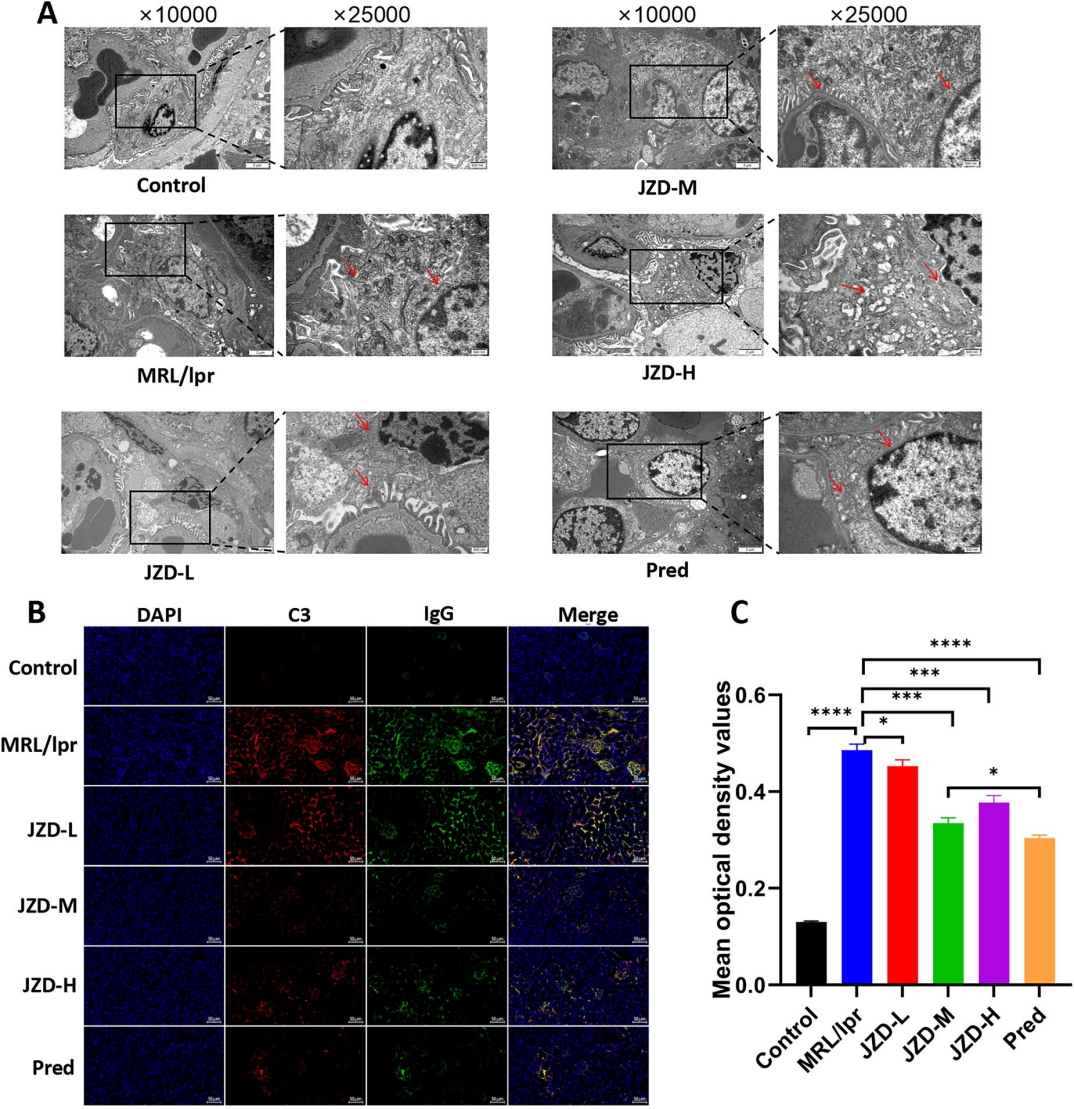
JZD attenuates IgG and C3 deposition in the kidneys of MRL/lpr mice. (a) The ultrastructure of glomeruli of MRL/lpr mice was observed by a TEM (left: 10000×, 2 μm; right: 25000×, 500 nm). (b) IgG (green) and C3 (red) deposition (20×, 50 μm) in the kidneys of MRL/lpr mice was determined by immunofluorescence. (c) The mean OD value of six groups was detected. *p < 0.05, ***p < 0.001,****p < 0.0001.

The ultrastructure of glomeruli was observed using a TEM. The results revealed that the mesangial cells in the control group showed multiple processes in the shape of stars, with the length of each process different, and the structure of dense patches and dense bodies could be seen. However, in the MRL/lpr group, there were electron dense deposits in the mesangial area, accompanied by mesangial cell proliferation and matrix increase. In contrast, electron dense deposition and mesangial cell proliferation were effectively reduced in the JZD-L, -M, and -H groups and the prednisone group compared with those in the MRL/lpr group (Figure 3(a)). Immunofluorescence was used to observe the deposition of IgG and C3 in the kidney, and it was found that the glomeruli in the control group were normal in size and clear in outline, and there was no obvious deposition of IgG and C3. However, the MRL/lpr group showed deposited IgG along the mesangial area in granules and clumps, and largely deposited C3 along the capillary wall and mesangial area. In contrast, the glomerular morphology and size tended to return to normal in the JZD-L, -M, and -H groups and the prednisone group, with alleviated IgG and C3 deposition to varying degrees (Figure 3(b, c)). These results indicated that JZD attenuated IgG and C3 deposition in the kidneys of MRL/lpr mice.

### Differential gene expression in SLE was analyzed and enrichment analysis was performed

**Figure 4.**
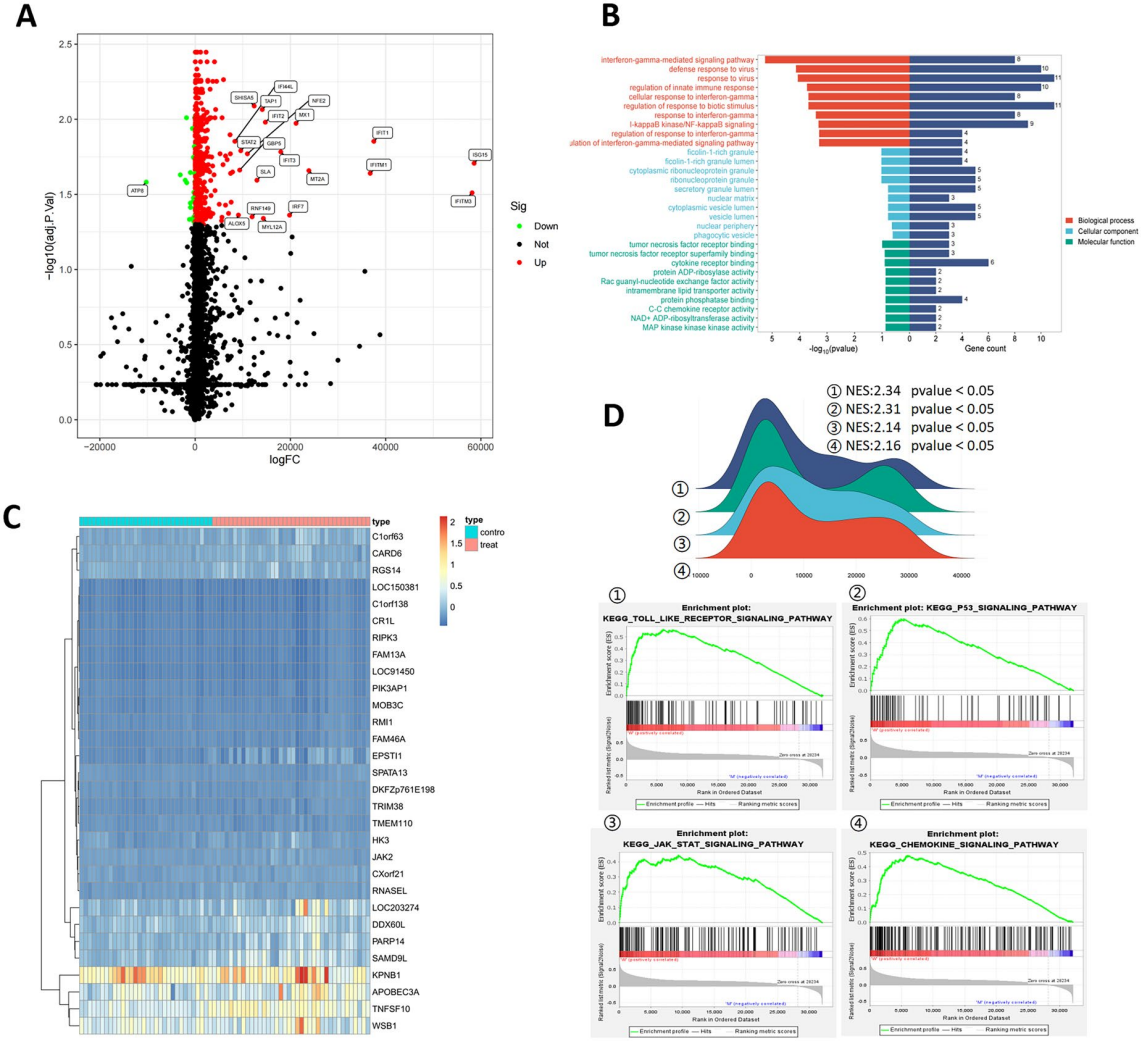
Differential gene expression in SLE is analyzed and enrichment analysis is conducted. (a) A volcano plot of DEGs was shown. (b) GO analysis was conducted on DEGs. (c) A heat map of differential gene expression results by RNA-seq was shown. (D) GSEA analysis was conducted on the whole dataset and the first four items were displayed.

Differential gene expression analysis was performed on the GSE154851 dataset of the GEO database. With the criteria of |*log*2FC|> 2 and *p*-value < 0.05, DEGs were screened, and 719 DEGs were obtained. The top 5 genes were TAP1, IRF7, STAT2, MYL12A, and MT2A (Figure 4(a, c)). The screening criteria were then set as |log2FC| > 4 and *p*-value < 0.01, resulting in 112 DEGs. These 112 DEGs were subjected to GO enrichment analysis, and it was found that in terms of biological process, genes were related to the regulation of innate immune response and cellular response to interferon-gamma; in terms of cellular component, genes were related to ribonucleoprotein granule, vesicle lumen, nuclear periphery, and phagocytic vesicle; in terms of molecular function, genes were associated with tumor necrosis factor receptor binding, protein phosphatase binding, and cytokine receptor binding (Figure 4(b)). Furthermore, GSEA enrichment analysis showed that this gene set was related to the chemokine signaling pathway, JAK/STAT pathway, P53 signaling pathway, and Toll-like receptor pathway (Figure 4(d)).

### Network pharmacology and molecular docking identified TLR4 as a potential target for JZD intervention in SLE

**Figure 5.**
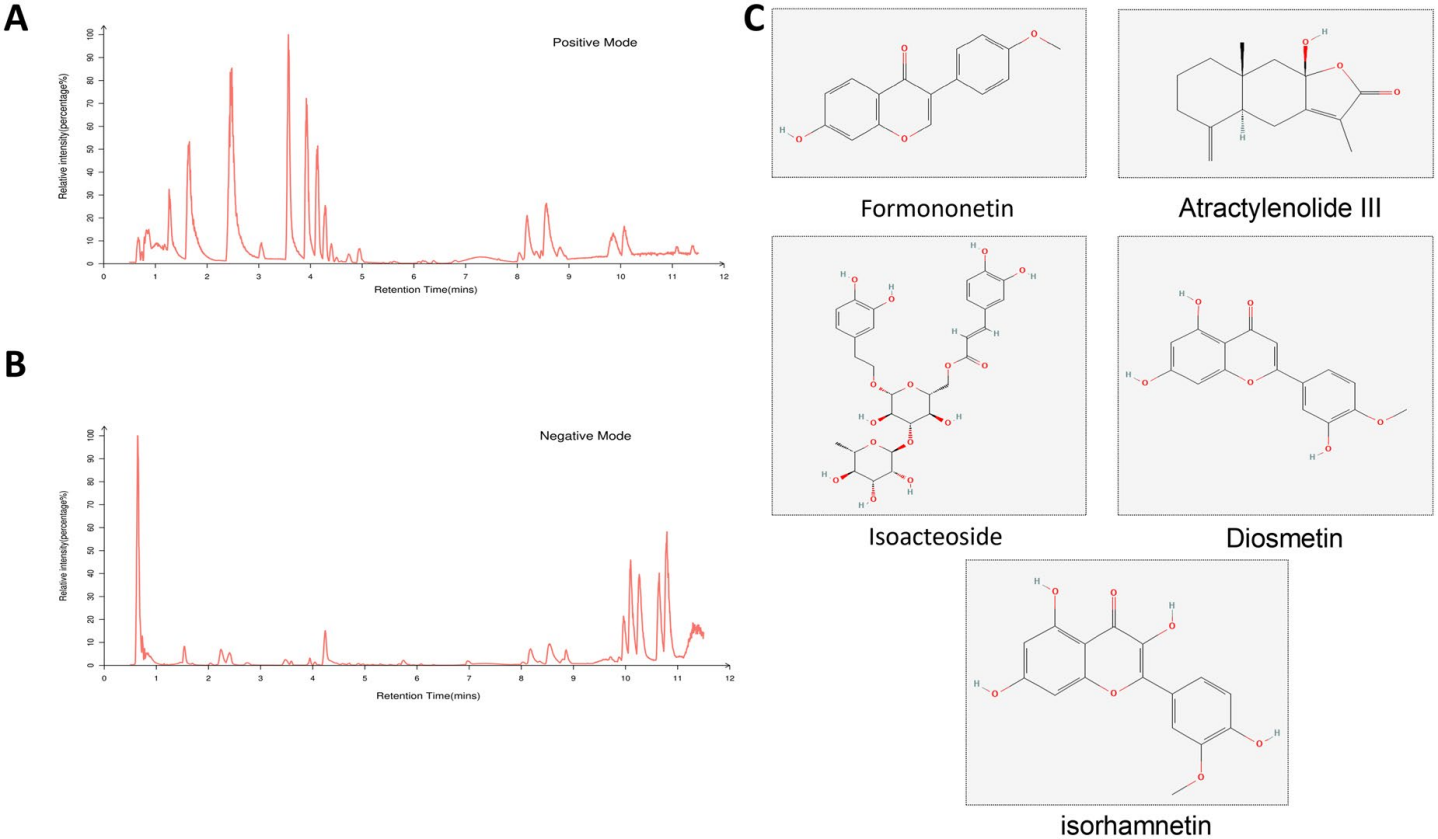
Base peak chromatogram (BPC) of JZD’s blood-entered components is displayed. (a) BPC in the positive ion mode was shown. (b) BPC in negative ion mode was shown. (c) The structural formula of the main compound of JZD.

**Figure 6.**
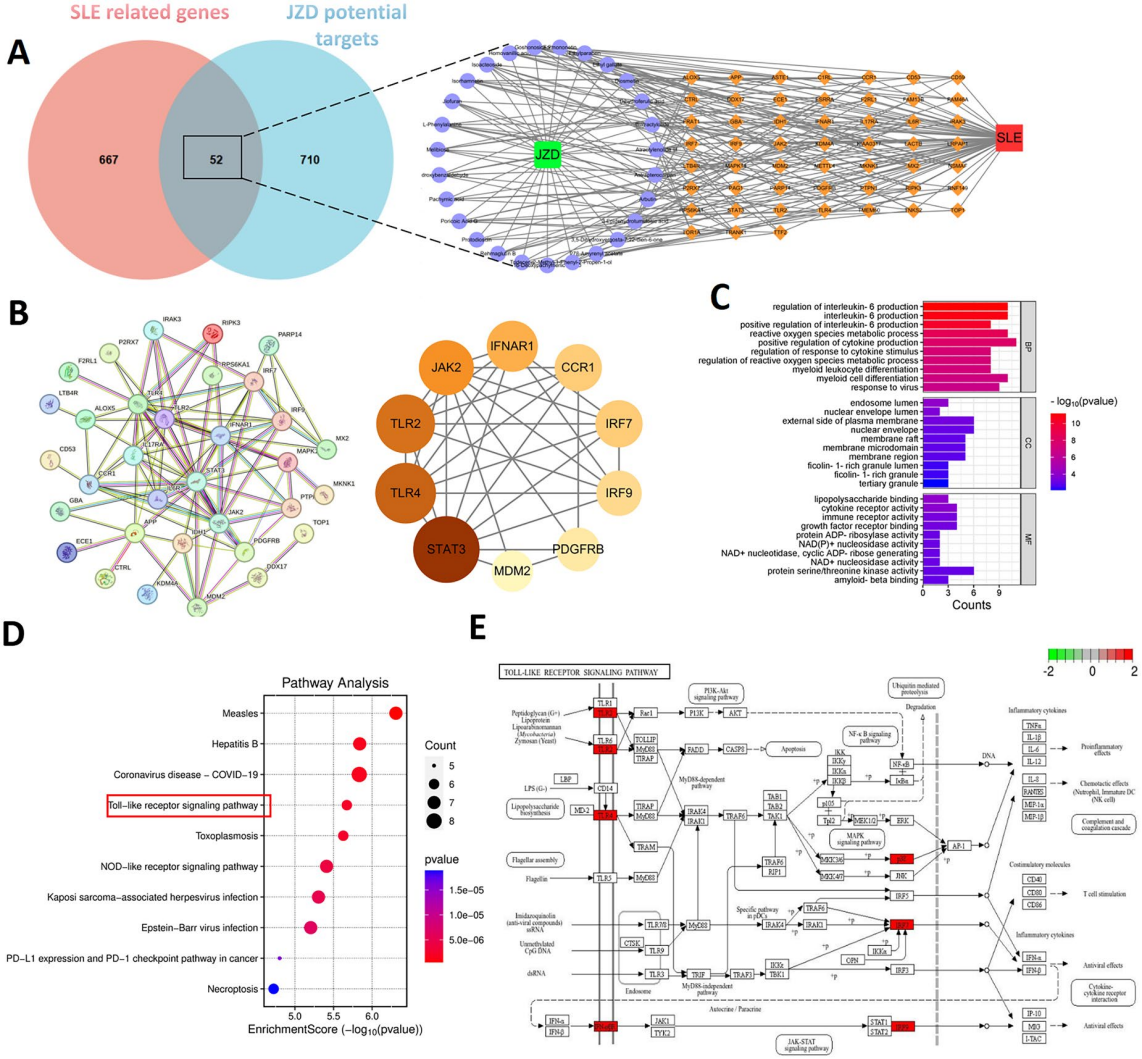
Network pharmacological analysis is conducted. (a) Venn diagram was used to show the intersection of potential targets of JZD’s blood-entered compounds and SLE-related genes to construct a ‘disease-component-target’ network diagram. (b) The PPI network diagram was constructed and key targets were identified. (c) GO enrichment analysis was conducted. (d) KEGG enrichment analysis was conducted. (e) The diagram of TLR signaling pathways was shown.

**Figure 7.**
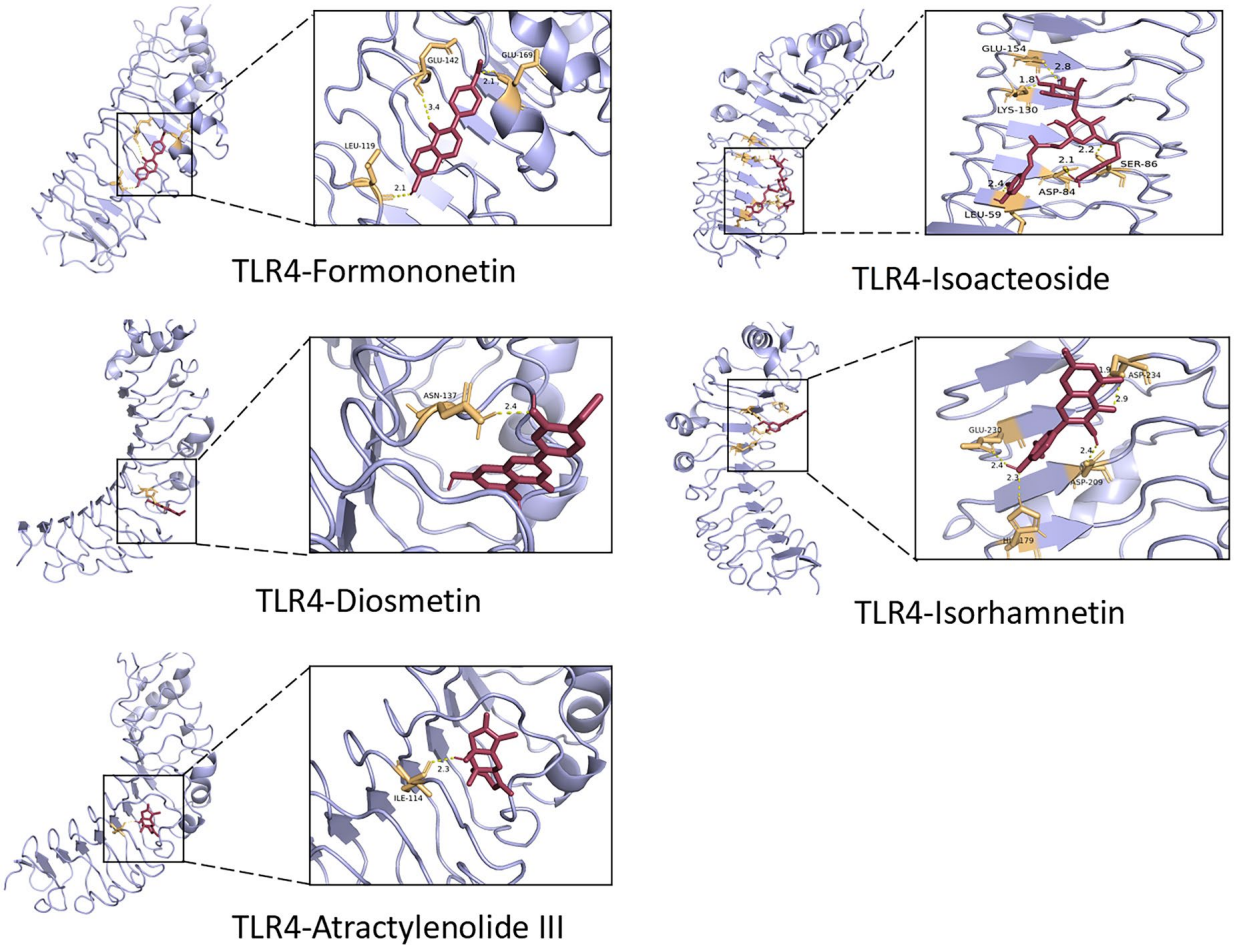
Molecular docking was conducted. The docking results of TLR4 with formononetin, isoacteoside, diosmetin, isorhamnetin, and atractylenolide III were visualized, respectively.

A total of 27 prototype components were identified in the blood-entered component of JZD based on UPLC-Q-TOF-MS/MS detection (Figure 5(a, b), Table S1) and literature reports. Next, 762 potential targets of 27 prototype components were predicted through the Swiss Target Prediction and HERB databases. Additionally, 719 DEGs were screened from the GEO database as targets of SLE. The potential targets of prototype components and DEGs were intersected and 52 intersection genes were obtained (Figure 6(a)). After that, the intersection genes were imported into the STRING database to construct a PPI network and subsequently identified TLR4 as the core target (Figure 6(b)). According to GO analysis results, in terms of biological process, it mainly involved regulation of interleukin-6 production, reactive oxygen species metabolic process, and positive regulation of cytokine production; in terms of cellular component, it was primarily enriched in nuclear envelope lumen, external side of the plasma membrane, and endosome lumen; in terms of molecular function, it mainly involved cytokine receptor activity, immune receptor activity, and lipopolysaccharide binding (Figure 6(c)). Furthermore, KEGG analysis results showed that it was closely related to the TLR signaling pathway (Figure 6(d, e)). Additionally, molecular docking results revealed that TLR4 had good binding ability with formononetin, isoacteoside, diosmetin, isorhamnetin, and atractylenolide III (binding energy ≤ −5 kcal/mol), and the results were visualized (Figure 7).

### JZD inhibited LPS-induced proliferation and inflammation in HMCs

**Figure 8.**
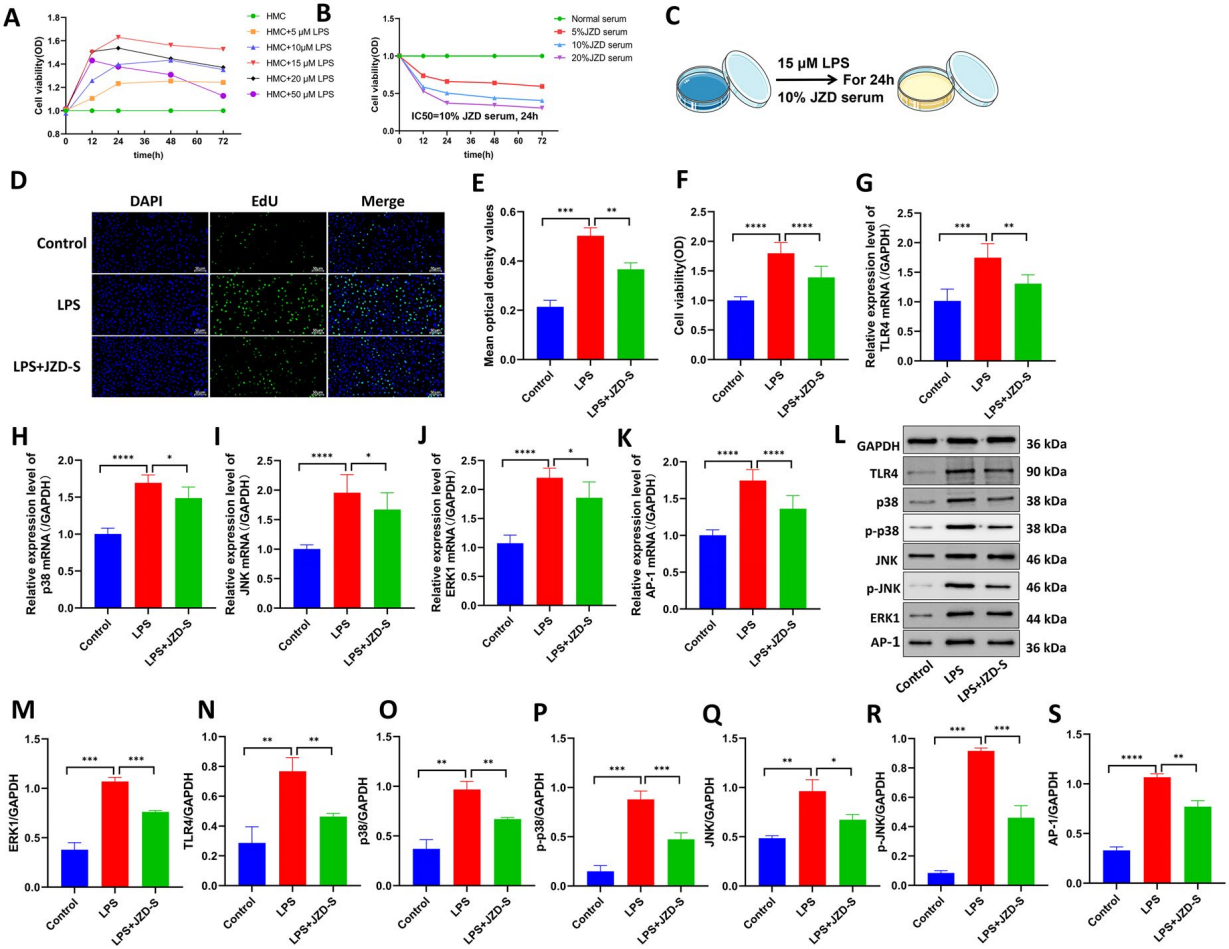
JZD inhibits LPS-induced proliferation of HMCs through the TLR4/MAPK pathway. (a) The optimal concentration of LPS for induction was screened. (b) The optimal serum concentration of JZD was screened. (c) The workflow of *in-vitro* experiments was shown. (d–e) EdU assay was used to detect the proliferation of cells in three groups (20×, 50 μm), with green fluorescence representing the positive EdU signal and blue fluorescence representing the nucleus. (f) The CCK-8 assay was used to assess cell viability in three groups. (g–k) RT-qRCR was used to detect the mRNA expression of TLR4, p38, JNK, ERK1 and AP-1 in three groups. (l–s) Western blotting was used to detect the protein level of TLR4, p38, p-p38, JNK, p-JNK, ERK1 and AP-1 in three groups (*n* = 3). *p < 0.05, **p < 0.01, ***p < 0.001, ****p < 0.0001.

**Figure 9.**
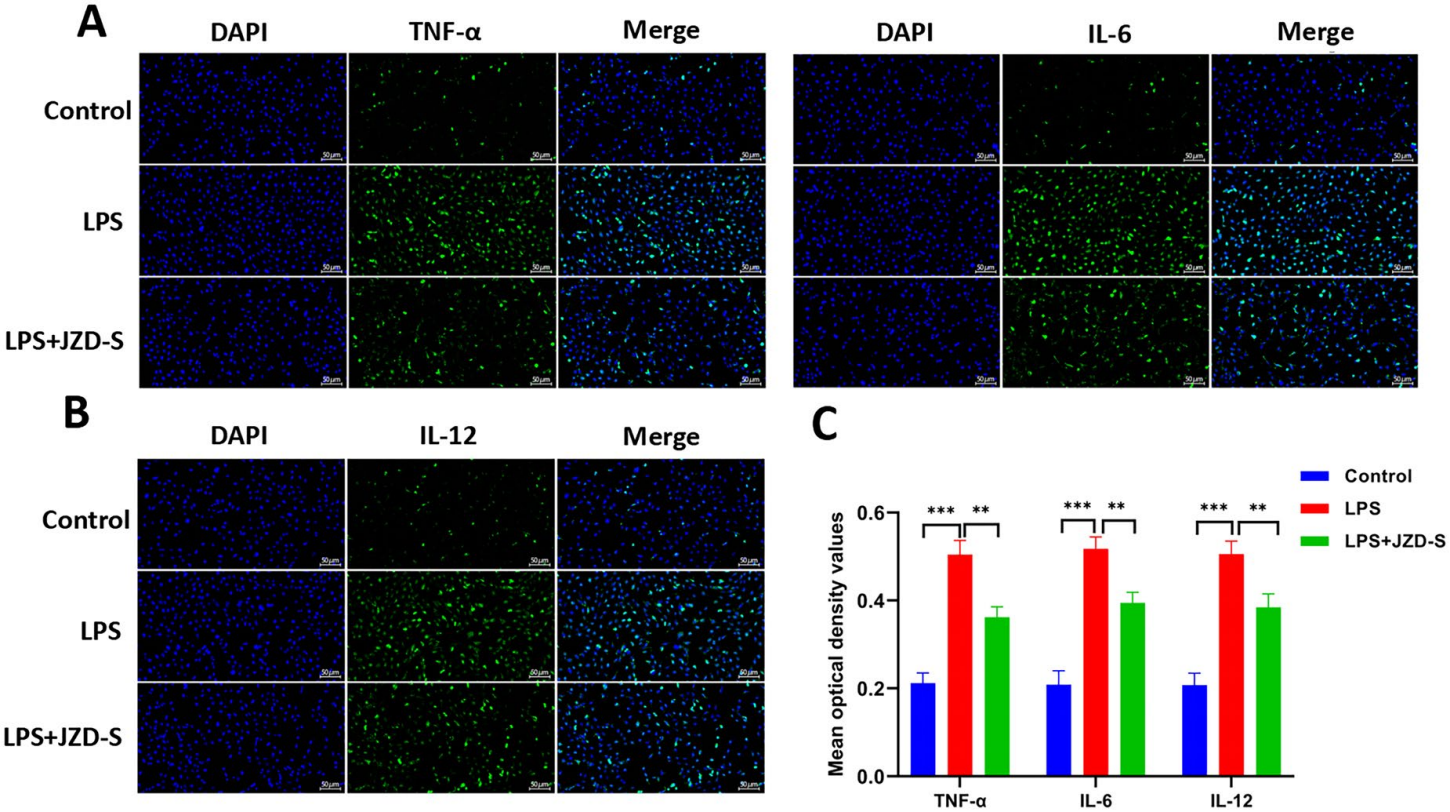
JZD inhibits LPS-induced expression of inflammatory factors in HMCs. (a, b) Immunofluorescence was used to detect the expression of TNF-α, IL-6, and IL-12 in the mesangial cells of three groups (20×, 50 μm). (c) The mean OD value of three groups was detected. **p < 0.01, ***p < 0.001.

Subsequently, the *in-vitro* experiments were conducted using HMCs to further explore the mechanism of JZD in improving renal damage. First, HMCs were induced with different concentrations of LPS (5 μM, 10 μM, 15 μM, 20 μM, and 50 μM) for 0 h, 12 h, 24 h, 48 h, and 72 h, respectively. The cell viability of each group was detected through CCK-8 assay. It was screened that the optimal induction concentration of LPS was 15 μM, and the action time was 24 h (Figure 8(a)). Second, HMCs were treated with 5%, 10%, and 20% JZD serum for 0 h, 12 h, 24 h, 48 h, and 72 h, respectively. The cell viability of each group was detected using CCK-8 assay. The results revealed that the optimal JZD serum concentration was 10% and the action time was 24 h (Figure 8(b)). The EdU method was used to detect the proliferation of HMCs in three groups. The results showed that compared with the control group, LPS induction notably increased the density of EdU-positive cells and enhanced the fluorescence signal. Compared with those in the LPS group, the density of EdU-positive cells was significantly reduced, and the fluorescence signal was weakened in the LPS + JZD-S group. This indicated that JZD serum inhibited mesangial cell proliferation (Figure 8(c–f)). Additionally, RT-qPCR results showed that TLR4, p38, JNK, and AP-1 mRNA expressions in the LPS group were increased relative to those in the control group. Compared with those in the LPS group, TLR4, p38, JNK, ERK1 and AP-1 mRNA expressions were decreased in the LPS + JZD-S group (Figure 8(g–k)). Furthermore, Western blotting results revealed that TLR4, p38, p-p38, JNK, p-JNK, ERK1 and AP-1 protein levels in the LPS group were higher than those in the control group, which were then notably reduced in the LPS + JZD-S group compared with those in the LPS group (Figure 8(l–s), *p* < 0.05). The above results indicated that JZD inhibited the expression of TLR4/MAPK pathway in mesangial cells. Additionally, immunofluorescence staining revealed that the LPS group exhibited increased TNF-α, IL-6, and IL-12 expressions and average fluorescence intensity compared with the control group. However, compared with the LPS group, the expression of TNF-α, IL-6, and IL-12 in the LPS + JZD-S group was decreased, and the average fluorescence intensity was decreased (Figure 9(a–c), *p* < 0.05). These results showed that JZD inhibited the inflammatory response of mesangial cells.

### JZD alleviated renal inflammation in MRL/lpr mice by mediating the TLR4/MAPK pathway

**Figure 10.**
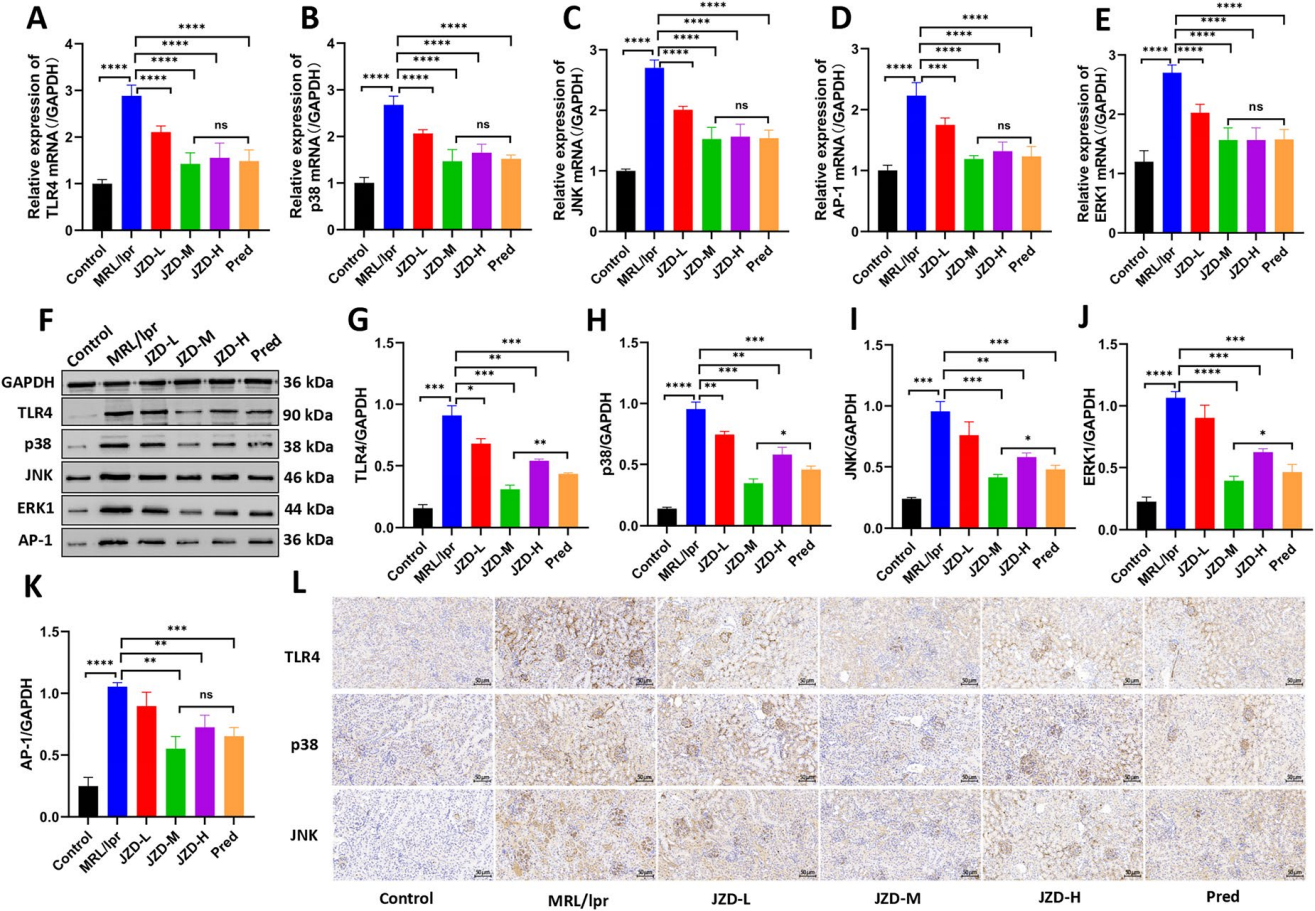
JZD inhibits the expression of the TLR4/MAPK pathway in MRL/lpr mouse kidneys. (a–e) RT-qRCR was used to detect the mRNA expression of TLR4, p38, JNK, ERK1 and AP-1 in the kidneys of mice in six groups. (f–k) Western blotting was used to detect the protein level of TLR4, p38, JNK, ERK1 and AP-1 in the kidneys of mice in six groups. (l) Immunohistochemical staining was used to detect the expression of TLR4, p38, and JNK in the kidneys of mice in six groups (20×, 50 μm), with brown indicating positive expression, and blue indicating the nucleus. *p < 0.05, **p < 0.01, ***p < 0.001, ****p < 0.0001.

**Figure 11.**
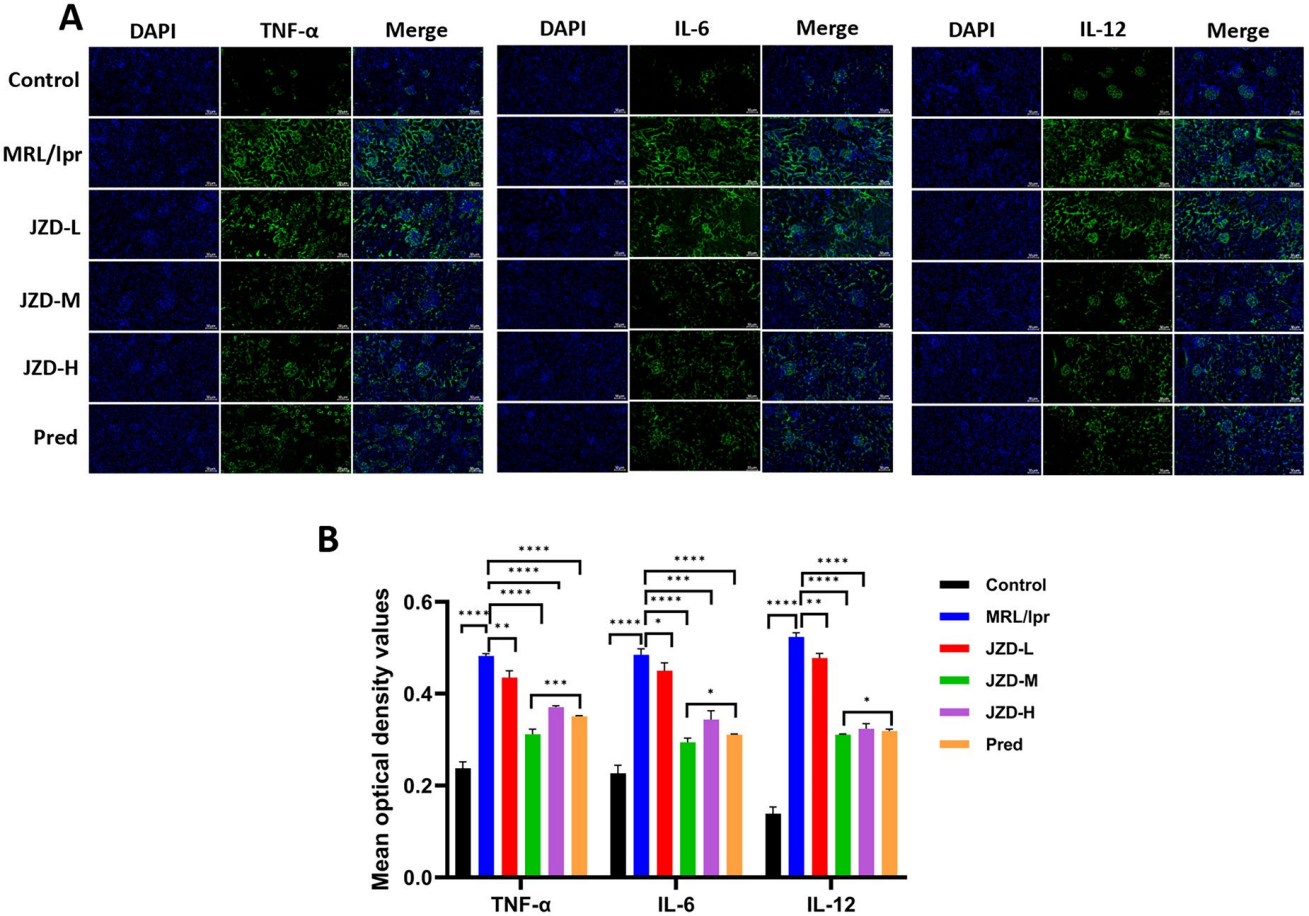
JZD inhibits the expression of inflammatory factors in the kidneys of MRL/lpr mice. (a) Immunofluorescence was used to detect the expression of TNF-α, IL-6, and IL-12 in the kidneys of mice in six groups (20×, 50 μm). (b) The mean OD value of six groups was detected. *p < 0.05, **p < 0.01, ***p < 0.001, ****p < 0.0001.

RT-qPCR and Western blotting were used to detect the expression of the TLR4/MAPK pathway-related molecules in the kidneys of MRL/lpr mice. The results showed that the mRNA expression and protein level of TLR4, p38, JNK, and AP-1 were increased in the MRL/lpr group compared with those in the control group. Compared with those in the MRL/lpr group, TLR4, p38, JNK, ERK1 and AP-1 mRNA expressions and protein levels were decreased in the JZD-L, -M, and -H groups and the prednisone group, especially in the JZD-M group (Figure 10(a–k), *p* < 0.05). Additionally, TLR4, p38, and JNK expressions in the kidney were detected through immunohistochemical staining. The results indicated that compared with that in the control group, the expression of TLR4, p38, and JNK in the MRL/lpr group was increased, and the positive signals were mainly distributed in the mesangial area and interstitium. Compared with the MRL/lpr group, JZD treatments (low-dose, medium-dose, and high-dose) and prednisone intervention could notably decrease the expression of TLR4, p38, and JNK in the kidneys, along with reduced distribution of positive signals, with medium-dose JZD showing a more obvious decreasing effects (Figure 10(l), *p* < 0.05). The results showed that JZD inhibited the expression of TLR4, p38, and JNK in the kidneys of MRL/lpr mice. Furthermore, the expression of TNF-α, IL-6, and IL-12 in the kidney was detected utilizing immunofluorescence staining, which was found to increase in the MRL/lpr group, along with increased mean fluorescence intensity, compared with the control group. Compared with those in the MRL/lpr group, TNF-α, IL-6, and IL-12 expressions were decreased and the mean fluorescence intensity was decreased in the JZD-L, -M, and -H groups and the prednisone group, especially in the JZD-M group (Figure 11(a, b), *p* < 0.05). Taken together, JZD reduced the level of renal inflammation in MRL/lpr mice. Moreover, the intervention effect of the medium-dose JZD is the best.

## Discussion

SLE is a heterogeneous disease with a high disability rate; SLE is still incurable at present with an unclear etiology, and its treatment methods mainly focus on controlling disease progression and intervening in target organ damage (Lazar and Kahlenberg 2023). In the diagnosis and treatment of SLE, assessment of disease activity is an important reference index for disease control and prognosis. A retrospective study has shown that anti-dsDNA antibody and 24h urinary protein levels were remarkably increased in SLE patients with hypocalcemia, while C3 and C4 levels and peripheral white blood cells and platelets were notably decreased, suggesting that serum calcium levels may be useful for assessing disease activity (Du et al. 2025). LN, a renal damage caused by SLE, is characterized by proteinuria and hematuria. Existing studies have demonstrated that the pathogenesis of LN is closely related to abnormal activation of innate immunity, immune complex deposition, and excessive activation of the downstream inflammatory signaling pathways; immune infiltration and production of inflammatory factors further aggravate renal inflammatory response (Bhargava et al. 2023; Chernova 2024).

The overall treatment goal for LN is to rapidly induce clinical remission and control proteinuria (Mok et al. 2025). For active LN, management guidelines have recommended glucocorticoids, mescaline, or low-dose intravenous cyclophosphamide as anchor drugs (Fanouriakis et al. 2024). Furthermore, in a clinical randomized controlled trial, LN patients treated with tacrolimus combined with intravenous cyclophosphamide have shown controlled urinary protein and disease activity and achieved clinical remission (Zheng et al. 2022). Failure to intervene promptly in the progression of renal damage may lead to poor patient prognosis and even death. Therefore, early screening for LN is important. As has been evidenced previously, anti-C1s antibodies are identical to anti-C1q antibodies as potential serologic biomarkers for early screening of LN (Vigne et al. 2025). Early clinical diagnosis and intervention is a fundamental guarantee for improving the prognosis of LN.

Although the application of glucocorticoids and immunosuppressants has improved the prognosis of SLE patients, there are many side effects, such as increased infection risk, metabolic disorders, and osteoporosis (Desai et al. 2024). Therefore, exploring safer and more effective alternative therapies (including TCM formulations) has become a focus of research. According to the TCM theory, the disease sites in LN are mainly in the ‘spleen’ and ‘kidney’. JZD used in this study is a TCM formula based on the disease site and has been widely used in clinical practice. In the present study, MRL/lpr mice were used as an animal model of lupus for *in-vivo* studies, and mice were administered with low-, medium-, and high-dose JZD, respectively. The results indicated that MRL/lpr mice highly mimicked the immune imbalance of SLE in humans. Additionally, JZD effectively reduced urinary protein, attenuated immune complex deposition in the kidneys, and ameliorated renal damage. Moreover, JZD could regulate the immune function of SLE patients, manifested as elevating C3 and C4 levels and decreasing anti-dsDNA antibody and IgG levels *in vivo*. To further explore the molecular mechanism of JZD’s action in SLE, we employed the GEO database and conducted a comprehensive analysis of network pharmacology. As a result, TLR4 was identified as one of the potential targets of JZD’s action, which was closely related to the TLR signaling pathway. Therefore, *in-vivo* experiments were further conducted, and the results demonstrated that JZD could inhibit the expression of TLR4, p38, JNK, ERK1 and AP-1 in the kidney, and reduce the level of inflammatory factors. And among different doses of JZD, the medium dose of JZD showed the best intervention effect.

It has been shown that aberrant activation of the TLR signaling pathway plays a central role in the pathogenesis of SLE (Bao et al. 2023). It can recognize endogenous nucleic acids and immune complexes, and drive pathogenic intrinsic immune activation, type I interferon storms, and self-reactive B cell activation, constituting the molecular basis of immune disorders in SLE (Dörner and Lipsky 2024; Lau et al. 2025). TLR4 acts as a pattern recognition receptor that drives pathological intrinsic immune responses in SLE by recognizing endogenous danger signals (HMGB1, nucleic acid complexes, etc.) and pathogen-associated molecular patterns [such as lipopolysaccharide (LPS)] (Hsieh et al. 2023). A previous study has shown that TLR4 expression is notably elevated on the surface of peripheral blood monocyte-derived macrophages and myeloid dendritic cells in SLE patients (Xiao et al. 2024). Inhibiting TLR4 expression can effectively control inflammation in MRL/lpr mice (Jing et al. 2022). TLR4 can trigger a series of downstream signaling and activate the MAPK family (including p38, ERK, JNK, etc.), which in turn induces the entry of transcription factors into the nucleus, mediates AP-1 expression, and promotes the secretion of pro-inflammatory factors (such as IL-6, TNF-α, IL-12, etc.), thus resulting in a persistent inflammatory cascade (Sundararaj et al. 2021; Lan et al. 2023). p38 MAPK enhances the pro-fibrotic effect of TGF-β1 on fibroblasts and promotes the synthesis of fibronectin, thereby accelerating renal interstitial fibrosis (Livingston et al. 2024). JNK upregulates nephrin protease expression in podocytes by phosphorylating c-Jun and disrupts the structure of the diaphragm, leading to proteinuria *In-vivo* studies found that the levels of pro-inflammatory factors (IL-6, TNF-α, and IL-12) were significantly reduced in the kidneys of MRL/lpr mice after JZD intervention (Sun et al. 2023). This finding was consistent with the results of previous studies.

To further validate TLR4 as a potential core target for JZD intervention in SLE kidney damage, we performed *in-vitro* cellular experiments. Specifically, HMCs were used as the subjects, and the lupus environment was induced using the TLR4 agonist LPS to construct a model of kidney damage *in vitro*. A previous study has proven that LPS can act as an inducer for the proliferation and inflammation in glomerular mesangial cells (Fan and Li 2024). The results of the present study indicated that when induced by LPS, HMCs exhibited notably increased proliferation and inflammation levels. JZD-contained serum could significantly inhibit the proliferation of HMCs and decrease the expression of TLR4, p38, JNK, ERK1 and AP-1, thus suppressing inflammatory responses. This study identified TLR4 as one of the key target genes of JZD’s action, and the TLR4/MAPK signaling pathway as the key regulatory pathway. Consistently, as has been evidenced previously, over-activation of TLR4 in the kidney exacerbates the inflammatory response and induces glomerulonephritis, which is closely related to the development of LN (Qi et al. 2020). The MAPK family, consisting of ERK1/2, p38 MAPK, and JNK, has emerged as a central hub for the pathological progression of LN by driving the inflammatory response, mesangial cell injury, and fibrosis process (Chai et al. 2024). It has been shown that p38 MAPK is widely activated in LN and promotes the synthesis and secretion of pro-inflammatory factors (IL-6, TNF-α, and IL-12). These cytokines attract and activate monocytes, macrophages, and neutrophils, leading to inflammatory cell infiltration. Additionally, p38 MAPK induces oxidative stress and leads to podocyte mitochondrial dysfunction and apoptosis, further exacerbating proteinuria (Woznowski et al. 2022). ERK1/2 activation promotes Th17 cell differentiation and increases the production of IL-17A, which further exacerbates inflammatory response. Additionally, ERK1/2 promotes mesangial cell proliferation and the expression of fibrosis-related genes (such as TGF-β1 and fibronectin) (Geng et al. 2020). Regarding the JAK/STAT pathway, the research group’s previous studies have found that JZD can also exert anti-inflammatory and anti-immune pharmacological effects by inhibiting the expression of JAK2 and STAT3. From all the above results, JZD could inhibit the expression of the TLR4/MAPK pathway-related factors, reduce the release of pro-inflammatory factors, and improve renal inflammatory response. JZD consists of complex and diverse components, and the synergistic effect of these active ingredients may constitute the material basis for the overall regulation of the immune inflammatory network by JZD.

Our results on the mechanism of JZD intervening in renal damage in SLE are highly consistent with previous studies. For example, a previous study has revealed that cordyceps protein attenuates the extent of renal damage in MRL/lpr mice by inhibiting the TLR4/MYD88/MAPK signaling pathway and mediating Th1 cell differentiation (Liao et al. 2024). Dihydroartemisinin alleviates SLE renal damage by regulating macrophage M1/M2 balance through the MAPK signaling pathway (Y. Chen et al., 2024). Notably, the present study for the first time directly correlates the efficacy of herbal compound with TLR4/MAPK pathway modulation, providing experimental evidence for the advantages of JZD multi-target intervention in SLE. Meanwhile, the results of this study have important implications for the clinical treatment of LN. For patients with hormone-dependent or resistant LN, JZD may be used as an adjunctive therapy to reduce the dosage of glucocorticoids by inhibiting the TLR4/MAPK pathway; for patients at subclinical stage, the early application of JZD may block the ‘inflammation-fibrosis’ vicious cycle and slow down the deterioration of renal function. Meanwhile, for patients using biological agents clinically, whether JZD can enhance the efficacy or improve the adverse reactions of biological agents is worthy of further study.

**Figure 12.**
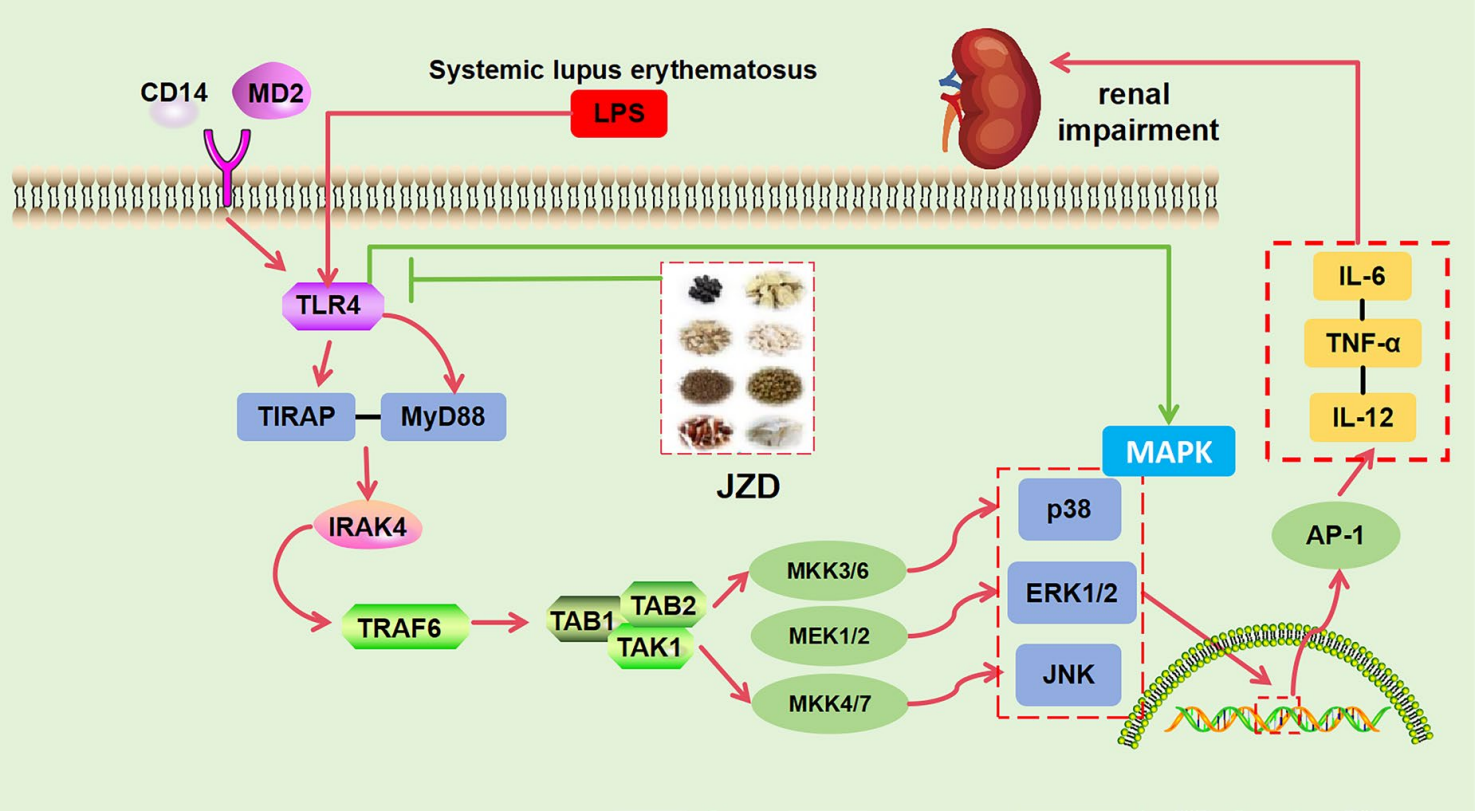
Mechanism diagram of JZD’s action in ameliorating renal damage in SLE through the TLR4/MAPK pathway.

In summary, JZD could inhibit the expression of TLR4, thus inhibiting the downstream over-activation of MAPK and the regulation of AP-1 transcription, reducing the release of pro-inflammatory factors, and exerting anti-inflammatory and anti-immune functions. The mechanisms identified in this study are summarized in Figure 12. However, there are still some limitations in this study. First, the interactions between the exact active ingredients and active substances of JZD have not been fully elucidated, requiring further pharmacological studies. In the future, we will further explore the mechanism of action of JZD through multi-omics analysis combined with single-cell sequencing technology. Meanwhile, multi-center clinical studies will be conducted to clarify the clinical efficacy and safety of JZD.

## Conclusions

Our study demonstrated that JZD could ameliorate immune complex deposition and attenuate mesangial cell proliferation and inflammation in SLE kidneys. Network pharmacology and molecular docking identified TLR4 as a potential target of JZD, which ameliorated renal inflammatory responses by inhibiting the downstream MAPK pathway. Subsequent multi-center and large-sample clinical trials will be conducted. These findings may provide a scientific basis for the clinical application of JZD and novel insights into the development of TLR4/MAPK dual-target inhibitors for SLE.

## Supplementary Material

supplementary tables.docx

## Data Availability

The datasets used and analyzed during the current study are available from the corresponding author on reasonable request.
