# Supplementary material for: Jianpi Zishen Decoction ameliorates renal damage induced by systemic lupus erythematosus through inhibition of the TLR4/MAPK pathway
Source: Pharm Biol. 2025 Dec 29;64(1):108–29. doi: 10.1080/13880209.2025.2606959 (PMC12777836; doi:10.1080/13880209.2025.2606959)
Supplement: supplementary tables.docx [file IPHB_A_2606959_SM3564.docx]

**Table S1** Compounds detected by UPLC-Q-TOF-MS/MS in positive and negative modes

| NO. | RT/min | Mode | Neutral mass  (Da) | Adducts | Observed  m/z | Calculated  m/z | Error (ppm) | Database Match Score (%) | Formula | Identified components |
| --- | --- | --- | --- | --- | --- | --- | --- | --- | --- | --- |
| 1 | 1.66 | Positive | 342.1162 | [M+Na]⁺ | 365.1015 | 365.1058 | 11.5 | 95 (mzCloud) | C_12_H_22_O_11_ | Melibiose |
| 2 | 1.69 | Positive | 196.0736 | [M+H]⁺ | 197.1024 | 197.1057 | 16.7 | 90 (GNPS) | C_8_H_8_O_4_ | Vanillic acid |
| 3 | 1.86 | Positive | 165.0790 | [M+H]⁺ | 166.0878 | 166.0863 | 9.0 | 98 (MassBank) | C_9_H_11_NO_2_ | L-Phenylalanine |
| 4 | 4.26 | Positive | 148.0888 | [M+H]⁺ | 149.0233 | 149.0211 | 14.8 | 88 (mzCloud) | C_10_H_12_O | 2-Methyl-1-Phenyl-2-Propen-1-ol |
| 5 | 5.32 | Positive | 182.0579 | [M+Na]⁺ | 205.0809 | 205.0793 | 7.8 | 93 (GNPS) | C_9_H_10_O_4_ | Homovanillic Acid |
| 6 | 7.05 | Positive | 624.2054 | [M+Na]⁺ | 647.8120 | 647.8105 | 2.3 | 92 (GNPS) | C_29_H_36_O_15_ | Isoacteoside |
| 7 | 7.76 | Positive | 316.0583 | [M+H]⁺ | 317.0700 | 317.0657 | 13.6 | 96 (mzCloud) | C_16_H_12_O_7_ | Isorhamnetin |
| 8 | 8.22 | Positive | 122.0368 | [M+H]⁺ | 123.0442 | 123.0441 | 0.8 | 99 (MassBank) | C_7_H_6_O_2_ | p-Hydroxybenzaldehyde |
| 9 | 12.30 | Positive | 300.1000 | [M+H]⁺ | 301.1429 | 301.1411 | 6.0 | 85 (GNPS) | C_17_H_16_O_4_ | Astrapterocarpan |
| 10 | 16.26 | Positive | 301.1394 | [M+H]⁺ | 302.0634 | 302.0647 | 4.3 | 94 (mzCloud) | C_16_H_12_O_6_ | Diosmetin |
| 11 | 18.37 | Positive | 236.0452 | [M+H]⁺ | 237.1451 | 237.1436 | 6.3 | 80 (GNPS) | C_9_H_13_ClO_5_ | Rehmaglutin B |
| 12 | 18.77 | Positive | 268.0736 | [M+H]⁺ | 269.1889 | 269.1863 | 9.7 | 93 (mzCloud) | C_16_H_12_O_4_ | Formononetin |
| 13 | 22.51 | Positive | 248.1412 | [M+H]⁺ | 249.1852 | 249.1824 | 11.2 | 97 (MassBank) | C_15_H_20_O_3_ | Atractylenolide III |
| 14 | 24.01 | Positive | 198.0528 | [M+H]⁺ | 199.1905 | 199.1873 | 16.1 | 91 (GNPS) | C_9_H_10_O_5_ | Ethyl Gallate |
| 15 | 26.61 | Negative | 484.3553 | [M+HCOO]⁻ | 529.3093 | 529.3058 | 6.7 | 87 (mzCloud) | C_31_H_50_O_3_ | 3-Epidehydrotumulosic Acid |
| 16 | 27.41 | Negative | 1048.5454 | [M-H]⁻ | 1047.5463 | 1047.5382 | 7.7 | 95 (MassBank) | C_51_H_84_O_22_ | Protodioscin |
| 17 | 27.95 | Negative | 462.2770 | [M+HCOO]⁻ | 507.3265 | 507.3225 | 7.9 | 89 (GNPS) | C_30_H_38_O_4_ | Biatractylolide |
| 18 | 27.98 | Negative | 528.3815 | [M-H]⁻ | 527.3569 | 527.3660 | 17.2 | 96 (mzCloud) | C_33_H_52_O_5_ | Pachymic Acid |
| 19 | 29.41 | Positive | 184.0736 | [M+H]⁺ | 185.0806 | 185.0810 | 2.2 | 91 (MassBank) | C_9_H_12_O_4_ | Jiofuran |
| 20 | 29.48 | Positive | 272.0896 | [M+H]⁺ | 273.0970 | 273.0970 | 0.0 | 98 (MassBank) | C_12_H_16_O_7_ | Arbutin |
| 21 | 29.65 | Positive | 1046.5656 | [M+H]⁺ | 1047.5764 | 1047.5764 | 0.0 | 82 (GNPS) | C_52_H_86_O_21_ | Protodioscin derivative |
| 22 | 30.65 | Negative | 182.2034 | [M+HCOO]⁻ | 227.2028 | 227.2019 | 4.0 | 84 (mzCloud) | C_13_H_26_ | 1-Tridecene |
| 23 | 31.07 | Positive | 428.3291 | [M+Na]⁺ | 451.3592 | 451.3587 | 1.1 | 92 (MassBank) | C_28_H_44_O_3_ | 3,5-Dihydroxyergosta-7,22-Dien-6-One |
| 24 | 31.17 | Positive | 468.3240 | [M+H]⁺ | 469.3792 | 469.3762 | 6.4 | 88(GNPS) | C_30_H_44_O_4_ | 16-Deoxypachymenic Acid B |
| 25 | 31.58 | Positive | 644.3408 | [M+H]⁺ | 645.4863 | 645.4836 | 4.2 | 92 (mzCloud) | C_32_H_52_O_13_ | Goshonoside F3 |
| 26 | 31.68 | Positive | 486.3345 | [M+H]⁺ | 487.3364 | 487.3345 | 3.9 | 89 (GNPS) | C_30_H_46_O_5_ | Poricoic Acid G |
| 27 | 31.78 | Positive | 468.3967 | [M+H]⁺ | 469.4059 | 469.4040 | 4.0 | 90 (mzCloud) | C_32_H_52_O_2_ | β-Amyrenyl Acetate |
